# Nonlinear relationship between HbA1c and coronary artery calcium score progression: a secondary analysis based on a retrospective cohort study

**DOI:** 10.1186/s13098-021-00747-z

**Published:** 2021-11-19

**Authors:** Jing Yu, Bo Gao

**Affiliations:** 1 Department of Radiology, The Affiliated Hospital of Guizhou Medical University, Guiyang, 550004 Guizhou Province China; 2 Key Laboratory of Brain Imaging, Guizhou Medical University, Guiyang, 550004 Guizhou Province China; 3 Department of Medical Imaging, Guizhou Medical University, Guiyang, 550004 Guizhou Province China

**Keywords:** Glycated hemoglobin, Coronary artery calcium score, Coronary atherosclerotic heart disease

## Abstract

**Objective:**

Coronary artery calcium score and glycated hemoglobin(HbA1c) are both considered risk factors for coronary heart disease. However, the relationship between coronary artery calcium score and HbA1c is still unclear. Consequently, the present study was undertaken to explore HbA1c association with coronary artery calcium score progression in South Korea.

**Methods:**

This study is a secondary analysis based on a retrospective cohort study in which 8151 participants received Health examination kits at the Health Promotion Center, Samsung Medical Center in Seoul, South Korea, from March 1, 2003–December 31, 2013. Cox proportional-hazards regression model was then used to evaluate the independent relationship between HbA1c and coronary artery calcium score progression.

**Results:**

After adjusting potential confounding factors (age, sex, BMI, height, weight, SBP, DBP, TC, LDL-C, HDL-C, triglycerides, smoking status, alcohol consumption, reflux esophagitis status, hypertension, diabetes, dyslipidemia, ischemic heart disease and cerebrovascular disease), it was revealed that there was a nonlinear relationship between HbA1c and coronary artery calcium score progression, while the scoring point was 5.8%. The effect size was 2.06 to the left of the inflection point, while the 95% CI was 1.85 to 2.29. Whereas, the effect size was 1.04, on the right side of the inflection point while 95% CI was 0.99 to1.10.

**Conclusion:**

The relationship between HbA1c and coronary artery calcium score progression is nonlinear. HbA1c is positively related to coronary artery calcium score progression when HbA1c level was less than 5.8%.

**Supplementary Information:**

The online version contains supplementary material available at 10.1186/s13098-021-00747-z.

## Introduction

Glycated hemoglobin (HbA1c) is the core of glycemic control and management in diabetic patients and has recently been recommended as a crucial parameter for diagnosing diabetes and identifying people at risk of developing it further [1]. HbA1c has a better reliability rate when it is compared with fasting or post-load blood glucose measurements [2]. HbA1c can also effectively reflect the blood glucose control achieved in the past 2–3 months and is a recommended tool for the diagnosis and screening of diabetes [3, 4].

Coronary atherosclerotic heart disease (CHD) is a multi-factorial disease. The Framingham Heart Study originally conducted in the US established a series of risk factors for coronary heart disease, including age, gender, cholesterol, hypertension, smoking, and diabetes [5]. It is a well-known fact that chronic glucose metabolism disorders increase the risk of CHD [6–9]. However, A series of evidence regarding the association between HbA1c and cardiovascular disease event outcome yields conflict findings. Many studies reported that HbA1c was an important determinant of CHD and its severity [10–14]. However, studies have reported that HbA1c level was associated with future cardiovascular risk in women without diabetes, while HbA1c level was not an independent determinant of cardiovascular risk in the population including women with diabetes [15]. Multiple observational studies and randomized trials have shown inconsistent evidence of the effect of HbA1c levels below current guidelines (7.0%) on cardiovascular events and death [16–19]. Given the differences in the study population, study design, measurement of coronary artery stiffness, adjustment for covariates, and certain methodological limitations, we conducted a secondary analysis based on a retrospective cohort study to observe the correlation between HbA1c and the progression of coronary artery calcium scores (CACS) in people undergoing physical examinations at the Health Promotion Center.

## Participants and methods

### Study design

This study had a retrospective cohort study design. The interesting independent variable in the present work is HbA1c, whereas the dependent variable is coronary artery calcium score progression (dichotomous variable: 0 = no progression, 1 = progression).

### Data source

We obtained data from the "PLOS ONE" database (https://journals.plos.org/plosone/). This website allowed users to download raw data for free. According to PLOS ONE Terms of Service, we cited PLOS ONE data package in the present study. (PLOS ONE data package: Min YW, Song BG, Kim HS, Kim K, Lee H, Min BH, Lee JH, Son HJ, Rhee PL, Kim JJ (2017) Data from: Associations between reflux esophagitis and the progression of coronary artery calcification: A cohort study. https://doi.org/10.1371/journal.pone.0184996.s001).

### Study population

Since the accomplishment of the entire study was achieved by Yang Won Min et al. [20], all the steps were outlined to understand the entire research process more clearly. Those patients were enrolled in a non-selective yet consecutive manner that had undergone physical examinations at the Health Promotion Center in Samsung Medical Center, Seoul, South Korea, from March 1, 2003, to December 31, 2013. Clinical data were extracted from the hospital electronic medical record system, and the patients' identities were encoded as non-traceable codes to ensure the complete privacy of all.

**Fig. 1:**
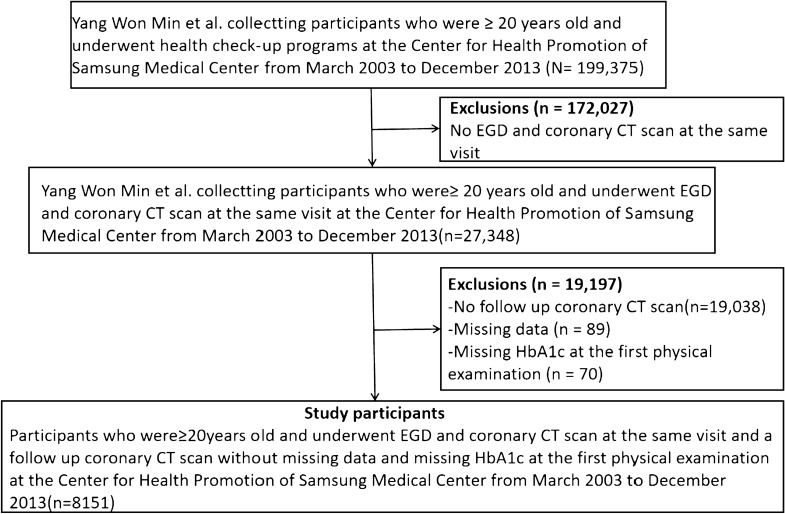
Study flow chart. *EGD* esophagogastroduodenoscopy, *CT*, computed tomography

Initially, 199,375 participants were involved in this study but later on, 191,224 participants were subsequently excluded, leaving 8151 cases for the final data analysis (Fig. 1). Inclusion criteria comprised of several factors like (1) Participants over 20 years who underwent health screening examinations; (2) Participants enduring the screening esophagogastroduodenoscopy (EGD) and coronary computed tomography (CT) scanning during the same visit. Whereas, the participants who did not undergo follow-up coronary CT scans (n = 19,038), and had missing information (n = 89) as well as skipped HbA1c at the first physical examination (n = 70) were excluded. Detailed ethics and informed consent was already ensured by the author in previously published articles [20].

### Variables

#### HbA1c

The information of HbA1c was obtained at baseline and was recorded as a continuous variable.

#### Coronary artery calcium score progression

Our interesting outcome variable was coronary artery calcium score progression (dichotomous variable: 0 = no progression, 1 = progression). Because there is no mutual definition for CACS progression in the literature, we used the transformed square root method of Hokanson to quantify the CACS progression recommended by John W et al. [21]. CACS progression was defined as the difference between the square root of the follow-up and baseline CAC scores ≥ 2.5. Brilliance 40 (Philips Medical Systems, Cleveland, Ohio), VCT Light Speed 64 (GE Healthcare, Milwaukee, Wisconsin), or Discovery 750HD (GE Healthcare) multidetector CT scanners were used to acquire images of CAC. The scans were analyzed using Extended Brilliance Workspace (Philips Medical Systems) or Advantage (GE Healthcare) workstations, while the CAC scores were calculated as described by Agatston et al. [22].

#### Covariates

In this study, the selection of covariates was based on the published literature and our accumulated clinical experience. Based on the above-mentioned principles, the following variables were used as covariates: (1) continuous variables: age, duration, body mass index (BMI), height, weight, systolic blood pressure (SBP), diastolic blood pressure (DBP), total cholesterol(TC), low-density lipoprotein cholesterol (LDL-C), high-density lipoprotein cholesterol (HDL-C), triglycerides; (2) categorical variables: sex (male, female), smoking status (never, past smoker, current smoker), alcohol consumption (never, past drinker, current drinker), reflux esophagitis status (negative, positive), hypertension (negative, positive), diabetes (negative, positive), dyslipidemia (negative, positive), ischemic heart disease (negative, positive), cerebrovascular disease (negative, positive).

### Statistical analysis

Data was indicated as mean ± standard deviation (normal distribution) or median (minimum, maximum)(skewed distribution) for continuous variables and as numbers and percentages for the categorical variables. χ2 (categorical variables), student t-test (normal distribution), or Mann–Whitney U test (skewed distribution) were used to detect the differences among different CACS progression (binary variables). To examine the association between HbA1c and CACS progression, three distinct models were constructed using univariate and multivariate Cox proportional-hazards regression model, including a non-adjusted model (no covariates were adjusted), minimally-adjusted model (only sociodemographic variables were adjusted) and fully-adjusted model (covariates present in Table 1 were adjusted). Effect sizes with 95% confidence intervals were recorded. Since Cox proportional-hazards regression model-based methods are often suspected for their inability to deal with non-linear models. For that reason, nonlinearity between HbA1c and CACS progression was addressed using the Cox proportional-hazards regression model with cubic spline functions and the smooth curve fitting (penalized spline method). If nonlinearity was detected, firstly, a calculation of the inflection point using a recursive algorithm was performed, and then a two-piecewise Cox proportional-hazards regression model was constructed on both sides of the inflection point (The details were described in Additional file 1).

**Table 1:** Baseline characteristics of participants (N = 8151)

	Coronary artery calcium score progression		
	No	Yes	P-value
N	3284	4867	
HbA1c, mean ± sd, (%)	5.51 ± 0.62	5.73 ± 0.82	< 0.001
Sex, (n, %)			0.012
Male	2956 (90.01%)	4460 (91.64%)	
Female	328 (9.99%)	407 (8.36%)	
Age group, (n, %)			< 0.001
< 65	3133 (95.40%)	4198 (86.25%)	
> = 65	151 (4.60%)	669 (13.75%)	
Duration, mean ± sd, (year)	3.69 ± 2.03	4.00 ± 2.23	< 0.001
BMI, mean ± sd, (kg/m^2^)	24.42 ± 2.48	24.94 ± 2.60	< 0.001
Height, mean ± sd, (cm)	169.84 ± 6.77	169.16 ± 6.59	< 0.001
Weight, mean ± sd, (kg)	70.61 ± 9.37	71.49 ± 9.51	< 0.001
SBP, mean ± sd, (mmHg)	117.59 ± 15.12	120.59 ± 15.83	< 0.001
DBP, mean ± sd, (mmHg)	74.92 ± 10.50	76.23 ± 10.52	< 0.001
TC, mean ± sd, (mg/dL)	197.69 ± 33.04	199.18 ± 34.87	0.085
LDL-C, mean ± sd, (mg/dL)	127.61 ± 30.05	128.77 ± 31.55	0.100
HDL-C, mean ± sd, (mg/dL)	52.60 ± 12.89	51.36 ± 12.51	< 0.001
Triglycerides, median (Q1-Q3), (mg/dL)	121.00 (86.00–171.00)	128.00 (93.00–181.00)	< 0.001
Smoking status, (n, %)			< 0.001
Never	960 (34.35%)	1201 (28.28%)	
Past smoker	998 (35.71%)	1755 (41.32%)	
Current smoker	837 (29.95%)	1291 (30.40%)	
Alcohol consumption, (n, %)			0.012
Never	399 (13.00%)	693 (14.98%)	
Past drinker	42 (1.37%)	85 (1.84%)	
Current drinker	2629 (85.64%)	3848 (83.18%)	
Reflux esophagitis status, (n, %)			0.001
Negative	2938 (89.46%)	4237 (87.06%)	
Positive	346 (10.54%)	630 (12.94%)	
Hypertension, n (%)			< 0.001
Negative	3097 (94.31%)	4423 (90.88%)	
Positive	346 (10.54%)	630 (12.94%)	
Diabetes, n (%)			< 0.001
Negative	3214 (97.87%)	4698 (96.53%)	
Positive	70 (2.13%)	169 (3.47%)	
Dyslipidemia, n (%)			0.012
Negative	3095 (94.24%)	4518 (92.83%)	
Positive	189 (5.76%)	349 (7.17%)	
Ischemic heart disease, n (%)			0.015
Negative	3279 (99.85%)	4844 (99.53%)	
Positive	5 (0.15%)	23 (0.47%)	
Cerebrovascular disease n (%)			1.000
Negative	3281 (99.91%)	4862 (99.90%)	
Positive	3 (0.09%)	5 (0.10%)	

*HbA1c* glycated hemoglobin, *BMI* body mass index, *SBP* systolic blood pressure, *DBP* diastolic blood pressure, *TC* total cholesterol, *LDL-C* low-density lipoprotein cholesterol, *HDL-C* high-density lipoprotein cholesterol, *CAC-P* coronary artery calcium score progression. The data presented are the means ± standard deviations, median (Q1–Q3) or numbers (percentages)

To test the robustness of our results, we performed a sensitivity analysis. We converted HbA1c into a categorical variable according to the quartile and calculated the *p* for the trend to verify the results of HbA1c as the continuous variable and to examine the possibility of nonlinearity.

To test whether the secondary prevention population would bias our results, we performed a sensitivity analysis. We excluded the secondary prevention population from the study and re-established a Cox proportional-hazards regression model to verify the stability of our results (adjustment variables were the same as fully adjused).

Modeling was performed with the statistical software packages R (http://www.R-project.org, The R Foundation) and EmpowerStats (http://www.empowerstats.com, X&Y Solutions, Inc., Boston, MA). The *p*-values less than 0.05 (two-sided) were considered statistically significant.

## Results

### Baseline characteristics of participants

The baseline characteristics of these included participants are listed in Table 1. The average age was 53.82 ± 7.66 years, and about 90.98% of participants were male. The incidence of progression was 59.71% (4867/8151). There were statistically significant differences between the no progression group and the progression group (*p-*values < 0.05) for all variables except total cholesterol and cerebrovascular disease (Table 1). When compared with no progression group, HbA1c, age, sex, duration, BMI, weight, SBP, DBP, TC, LDL-C, triglycerides, smoking status, alcohol consumption, reflux esophagitis status, hypertension, diabetes, dyslipidemia, ischemic heart disease and cerebrovascular disease increased significantly in the progression group, while the opposite results were detected in covariates in terms of height and HDL-C.

### The results of multivariate analyses using Cox proportional-hazards regression model

To evaluate the correlation between HbA1c and CACS progression, three different models (Cox proportional-hazards regression model) were constructed (Table 2). In the un-adjusted model, an increase of 1% of HbA1c was related to a 24% increase of CACS progression risk (HR = 1.24, 95%CI: 1.21 to 1.28), leading to a statistically significant result. In the minimally-adjusted model, adjustment of demographic variables led to an additional increase of 1% of HbA1c by 23% (HR = 1.23, 95%CI: 1.20 to 1.27). In fully-adjusted model, each additional 1% of HbA1c was accompanied by a 22% increases in CACS progression (HR = 1.22, 95%CI:1.18 to 1.26). The distribution of confidence intervals indicated that the correlation between HbA1c and CACS progression obtained by the model was reliable.

**Table 2:** Univariate and multivariate analyses of the association between HbA1c and coronary artery calcium score progression

Variable	Non-adjusted model HR (95%CI)	Minimally-adjusted model HR (95%CI)	Fully-adjusted model HR (95%CI)
HbA1c (%)	1.24 (1.21, 1.28)	1.23 (1.20, 1.27)	1.22 (1.18, 1.26)
HbA1c group			
Q1	1.0	1.0	1.0
Q2	1.30 (1.19, 1.42)	1.29 (1.18, 1.41)	1.39 (1.26, 1.53)
Q3	1.60 (1.46, 1.75)	1.56 (1.43, 1.71)	1.62 (1.46, 1.79)
Q4	2.02 (1.85, 2.20)	1.93 (1.76, 2.10)	1.97 (1.79, 2.17)
P for trend	< 0.0001	< 0.0001	< 0.0001

*HR* Hazards ratio, *CI* confidence interval, *HbA1c* glycated hemoglobin

Non-adjusted model: we do not adjusted for any variates

Minimally-adjusted model: only sex and age are adjusted for Fully-adjusted model: all variates presented in Table 1 are adjusted for

### The nonlinearity addressed by Cox proportional-hazards regression model with cubic spline functions

**Fig. 2:**
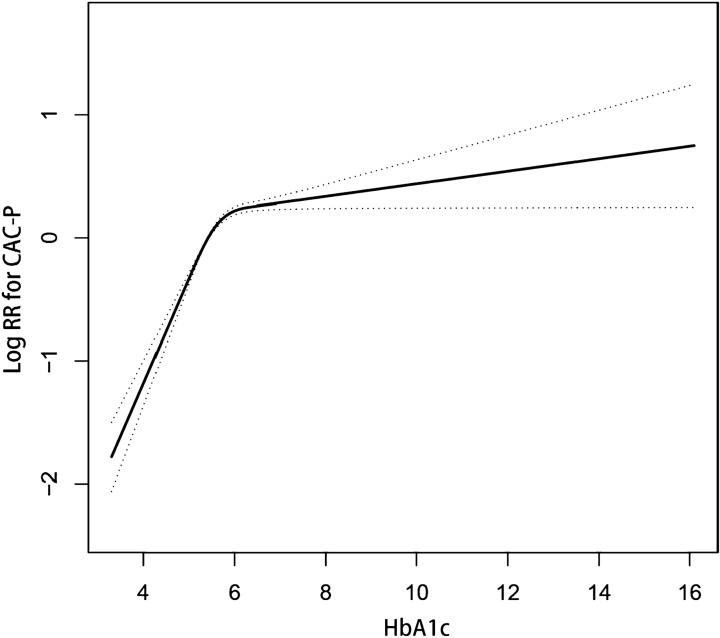
Relationship between HbA1c and coronary artery calcium score progression by using Lowess smoothing technique. *HbA1c* glycated hemoglobin, *CAC-P* coronary artery calcium score progression

Usage of Cox proportional-hazards regression model along with cubic spline functions and a smooth curve fitting, it was observed that the correlation between HbA1c and CACS progression was nonlinear (Fig. 2) due to which the data was adjusted proportionally to a piecewise Cox proportional-hazards regression model to fit two different slopes. Based on the sensitivity analysis, data was also arranged by standard Cox proportional-hazards regression model, and the best fit model was selected through log-likelihood ratio test (Table 3). As the *p*-value for the log-likelihood ratio test was less than 0.05 in our study, the two-piecewise model was utilized to fit the relationship between HbA1c and CACS progression. By using a recursive algorithm, firstly, the inflection point was discovered as 5.8, and then the effect sizes and confidence interval around the inflection point were computed through a two-piecewise Cox proportional-hazards regression model. To the left of the inflection point, the effect size was 2.06, and the 95% CI was 1.85 to 2.29, whereas the effect size was 1.04, and the 95% CI was 0.99 to1.10 on the right side of the inflection point.

**Table 3:** Nonlinearity explaination on HbA1c and coronary artery calcium score progression using two-piecewise linear model

	Effect size (95%CI) P value
Fitting model by standard linear regression	1.22 (1.18, 1.26) < 0.0001
Fitting model using two-piecewise linear model	
Inflection point	5.8
< 5.8	2.06 (1.85, 2.29) < 0.0001
> 5.8	1.04 (0.99, 1.10) 0.1179
P for log-likelihood ratio test	< 0.001

The adjustment strategy is the same with fully-adjusted model

## Discussion

In this observational retrospective cohort study, it was concluded that HbA1c and CACS progression have a nonlinear saturated relationship. When the HbA1c level was less than 5.8%, a positive correlation was observed between HbA1c and the progression of CACS, while above 5.8%, this positive correlation gravitated towards saturated. Even if HbA1c levels increased again, the risk of CACS progression would not surge ahead. There was no significant alteration after excluding the secondary prevention population in the study (Additional file 1: Table S1).

Coronary artery calcification score is a strong predictor of cardiovascular disease events [23]. The progression of CACS has been associated with a higher risk of myocardial infarction and all-cause mortality [24–26]. In populations with other risk factors for cardiovascular disease events (e.g., hypertension, dyslipidemia, smoking, etc.), more aggressive control of HbA1c levels in those with HbA1c below 5.8 may have greater potential benefits. When HbA1c is higher than 5.8, the change in HbA1c level may have a scarce effect on the outcomes of cardiovascular events.

Some previous studies have shown consistent results with ours investigating correlation between HbA1c and CACS progression or coronary atherosclerosis. Reza Ajudani, in a cross-sectional study based on 411 patients without history of known diabetes mellitus, reported that HbA1c may be functioning as an independent diagnostic factor in nondiabetic patients with severe coronary atherosclerosis [27]. Besides, Wenhui Zhao et al. used African American and white diabetic patients as the study population and obtained similar results [28]. Rivera et al. reached similar conclusions in 1043 asymptomatic people without diabetes [29]. Additionally, Peter D Reaven et al., in a study comprising of veteran diabetic participants, stated that although intensive hypoglycemic therapy did not reduce cardiovascular events in the study cohort as a whole, an intensified hypoglycemic therapy could reduce cardiovascular events in people with less severe coronary atherosclerosis [30]. In a large enrollment study, the authors found that lower levels of HbA1c, systolic blood pressure and LDL were associated with a reduced risk of acute myocardial infarction and stroke. In patients with type 2 diabetes, the HbA1c level outside the target range recommended in current guidelines (7%) was a strong predictor of cardiovascular event outcomes [31]. This is closely related to our results.

However, the reduction of HbA1c level is not completely related to the occurrence and improvement of cardiovascular events. Hertzel C Gerstein et al. reported that using intensive therapy to position glycosylated hemoglobin levels below 6.0% could increase mortality, but did not significantly reduce major cardiovascular events in type 2 diabetes [32]. The inconsistency of these studies may be due to the small sample size, different study populations, different forms of outcome variables, and few cases of coronary heart disease, which may limit the statistical power.

Our study has some innate strength that is listed below. Firstly, the sample size of this study was relatively large. Secondly, all the previously cited articles that also studied HbA1c and CACS or coronary atherosclerosis did not clarify the nonlinear relationship, which we have tried to highlight in our study [27, 28, 33–38]. To the best of our knowledge, this is the first time that a nonlinear relationship has been reported, along with the determination of an inflection point exploring the correlation between HbA1c and CACS progression. Third, this study is an observational study and therefore susceptible to potential confounding, although we used strict statistical adjustment to minimize residual confounders. Lastly, we ensured the robustness of the results through sensitivity analysis (conversion of target-independent variable form and exclusion of secondary prevention population) which made our results more reliable.

Our research also has few shortcomings which need attention. Firstly, our findings can be generalized only to persons who had undergone physical examinations in South Korea. Secondly, we could not observe the correlation between HbA1c and the progression of CACS in patients with diabetes and prediabetes, which can be attributed to the limitation of the original data. Similarly, we could also not consider fasting blood glucose as a confounding factor to perceive the relationship between HbA1c and CACS progression. In addition to that, there was no differentiation between the patients whose variables were in the abnormal range without any specific intervention, either partial or complete and patients who achieved.

the observed normal levels with medication. Last but not least, we can only adjust the measurable confounding factors, but not the unmeasurable confounding factors.

## Conclusion

The relationship between HbA1c and coronary artery calcium score progression is nonlinear. HbA1c is positively related to coronary artery calcium score progression when HbA1c level was lower than 5.8%.

## Supplementary Information

**Additional file 1: Additional methods.** Statistical analysis: the calculation method of inflection point. **Table S1.** The results of HR with and without secondary prevention populations.

## Data Availability

Data can be downloaded from the "PLOS ONE" database (https://journals.plos.org/plosone/).

## Abbreviations

HbA1c: Glycated hemoglobin
CHD: Coronary heart disease
BMI: Body mass index
SBP: Systolic blood pressure
DBP: Diastolic blood pressure
TC: Total cholesterol
LDL-C: Low-density lipoprotein cholesterol
HDL-C: High-density lipoprotein cholesterol
CACS: Coronary artery calcium score progression
EGD: Esophagogastroduodenoscopy
CT: Coronary computed tomography
