# Supplementary material for: Nonlinear relationship between HbA1c and coronary artery calcium score progression: a secondary analysis based on a retrospective cohort study
Source: Diabetol Metab Syndr. 2021 Nov 19;13:136. doi: 10.1186/s13098-021-00747-z (PMC8603599; doi:10.1186/s13098-021-00747-z)
Supplement: Supplementary file 1 — Additional file 1: Additional methods. Statistical analysis: the calculation method of inflection point. Table S1. The results of HR with and without secondary prevention populations. [file 13098_2021_747_MOESM1_ESM.docx]

**Additional methods**

**Statistical analysis: the calculation method of inflection point**

We first use smooth curve fitting to examine whether the independent variable is partitioned into intervals. We apply segmented regression (also known as piece-wise regression) that is using a separate line segment to fit each interval. Log-likelihood ratio test comparing one-line (non-segmented) model to segmented regression model was used to determine whether threshold exists. The inflection point that connecting the segments was based on the model gives maximum likelihood, and it was determined using two steps recursive method.

The step 1 is to narrow down the inflection point to a 10 percentile range of the independent variable.  From 5% to 95% incremented by 5%, we test 19 segmented regression models using these 19 percentile points of independent variable as the inflection point respectively to find out which percentile points gives the model with highest likelihood. The precise inflection point was narrowed down to +/- 4% percentile of the percentile points which gives highest likelihood among the 19 models, called Kmin and Kmax respectively.

The step 2 is to determine precise inflection point between Kmin and Kmax using the recursive method. The specific method is to first run 3 models with inflection point equals Q1 (25% percentile), Q2 (50% percentile) and Q3 (75% percentile) within the range of Kmin and Kmax respectively to find out which quartile point gives the model with highest likelihood among the three models. Then we narrow down the Kmin and Kmax to the range of +/- 25% of the corresponding quartile point. By doing so, we narrow down the range of Kmin and Kmax 50% recursively each time until the specific value of the independent variable was identified, that if used as inflection point will give the segmented regression model highest likelihood.

**Additional file 1: Table S1: The results of HR with and without secondary prevention populations**

| Variable | Fully-adjusted model I  HR (95%CI) | Fully-adjusted model II  HR (95%CI) |
| --- | --- | --- |
| HbA1c (%) | 1.22 (1.18, 1.26) | 1.28 (1.22, 1.33) |
| HbA1c group |  |  |
| Q1 | 1.0 | 1.0 |
| Q2 | 1.39 (1.26, 1.53) | 1.38 (1.22, 1.56) |
| Q3 | 1.62 (1.46, 1.79) | 1.48 (1.30, 1.68) |
| Q4 | 1.97 (1.79, 2.17) | 1.96 (1.74, 2.22) |
| P for trend | <0.0001 | <0.0001 |

Fully adjusted model I: The study included secondary prevention populations such as hypertension, diabetes, dyslipidemia, ischemic heart disease, and cerebrovascular disease. We adjusted age, sex, BMI, height, weight, SBP, DBP, TC, LDL-C, HDL-C, triglycerides, smoking status, alcohol consumption, reflux esophagitis status, hypertension, diabetes, dyslipidemia, ischemic heart disease and cerebrovascular disease.

Fully adjusted model II:Secondary prevention populations ( hypertension, diabetes, dyslipidemia, ischemic heart disease, and cerebrovascular disease ) were excluded in the study. We adjusted age, sex, BMI, height, weight, SBP, DBP, TC, LDL-C, HDL-C, triglycerides, smoking status, alcohol consumption and reflux esophagitis status.

HR:hazard ratio

CI: confidence interval
